# Development of an inexpensive, simple purification method for ω-5 gliadin from wheat flour^✰^

**DOI:** 10.1016/j.mex.2026.103979

**Published:** 2026-06-01

**Authors:** Akihiro Maeta, Mamoru Tanaka, Kyoko Takahashi

**Affiliations:** a Department of Food Science and Nutrition, Mukogawa Women’s University, Nishinomiya, Hyogo, Japan; b College of Bioscience and Biotechnology, Chubu University, Kasugai, Aichi, Japan

**Keywords:** ω-5 gliadin, Wheat, Purification, Acid-polyacrylamide gel electrophoresis (A-PAGE), Electroelution

## Abstract

ω-5 gliadin is a major causative allergen associated with various types of wheat allergy, including wheat-dependent exercise-induced anaphylaxis. Recently, ω-5 gliadin-deficient wheat has been developed. However, several wheat protein studies do not use standard ω-5 gliadin because its naturally derived form is expensive, and purifying it in-house is time-consuming. Therefore, we sought to develop an inexpensive, simple method for purifying ω-5 gliadin on a small scale.

▪ We compared ω-5 gliadin-deficient wheat with normal wheat and screened for ω-5 gliadin bands using acid-polyacrylamide gel electrophoresis (A-PAGE) on a mini-slab electrophoresis apparatus.
▪ The ω-5 gliadin band was excised and sealed in dialysis tubing, and the protein was electroeluted using agarose gel electrophoresis. After electroelution, the eluted solution was filtered, dialyzed, and then freeze-dried.
▪ The main band of collected proteins migrated at approximately 55 kDa in SDS-PAGE, and the most intense band was identified as ω-5 gliadin using nano-LC-MS/MS.

## Graphical abstract

**Figure:**
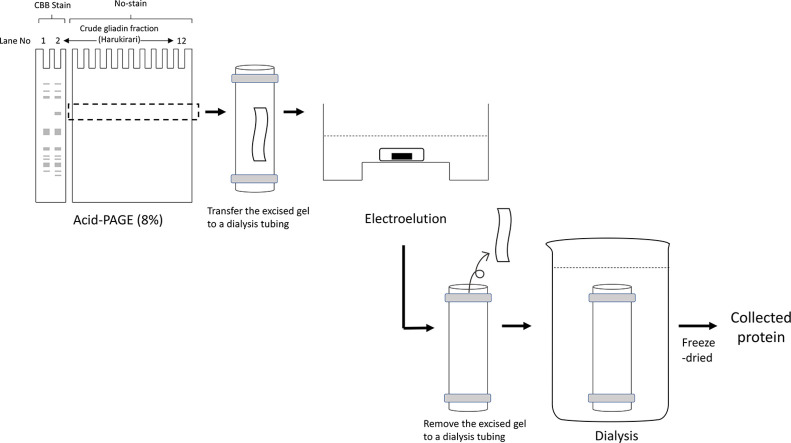

**Specifications table**

**Table:** 

**Subject area**	Agricultural and Biological Sciences
**More specific subject area**	Wheat allergy
**Name of your method**	Simple method for purifying ω-5 gliadin
**Name and reference of original method**	H. Watry, A. Zerkle, D. Laudencia-Chingcuanco, Modified acid-PAGE method for rapid screening and phenotyping of wheat gliadin mutant lines. *MethodsX* (2020) 7 100,858. https://doi.org/10.1016/j.mex.2020.100858.
**Resource availability**	

## Background

Gliadin and glutenin are the main wheat storage proteins. Glutenins are categorized further into high- and low-molecular weight subunits, while gliadins are classified into ω-, γ-, and α/β-gliadins [[1], [2], [3]]. This classification is based on their mobilities in acid-polyacrylamide gel electrophoresis (A-PAGE) [[1], [2], [3], [4], [5], [6]]. Among these gliadins, ω-5 gliadin has attracted particular attention. ω-5 gliadin is a major causative allergen for various types of wheat allergy, including wheat-dependent exercise-induced anaphylaxis (WDEIA) [3,[7], [8], [9], [10]]. However, reducing the allergenicity of ω-5 gliadin using food processing methods commonly used in households is challenging. In an attempt to reduce the allergenicity of ω-5 gliadin, ω-5 gliadin-deficient wheat has been developed [2,7,10,11].

Extensive research on ω-5 gliadins has been conducted; however, several wheat protein studies still do not use standard ω-5 gliadin [2,[4], [5], [6],[10], [11], [12]]. The proportion of ω-gliadins in wheat gliadin is lower than that of γ- and α/β-gliadins, and ω-gliadins are further divided into ω-1,2 gliadin and ω-5 gliadin [[2], [3], [4], [5]]. Therefore, the amount of ω-5 gliadin in wheat flour is very low, and ω-5 gliadin derived from natural sources is expensive (approximately $3000/50 μg; Biostir Inc., Osaka, Japan). Methods for purifying ω- or ω-5 gliadin from natural sources have been reported, including those using the PrepCell Model 491 electrophoretic separation system (Bio-Rad Laboratories, Hercules, CA, USA) [4,5], reversed-phase high-performance liquid chromatography (RP-HPLC) [1,7], and gel column chromatography [3,9]. PrepCell Model 491 (Bio-Rad Laboratories) is an uncommon and expensive laboratory equipment, and chromatographic methods require several laborious and complicated steps. Here, we hypothesized that ω-5 gliadins could be purified inexpensively and simply by excising gel bands from A-PAGE and electroeluting proteins from the band. Accordingly, we aimed to develop an inexpensive, simple method for the small-scale purification of ω-5 gliadin.

## Method details

### Materials and reagents

‘Harukirari’ (Bakerista Mills Corp, Hokkaido, Japan), ‘Norin 61′ (Maeda Shokuhin Co., Ltd., Saitama, Japan) and ω-5 gliadin-deficient wheat (Medical Kosho Co., Ltd., Shimane, Japan) were prepared as experimental wheat flour. ‘Harukirari’ is a fine flour and often used to sandwich bread, Danish pastries and croissants. ‘Norin 61’ is a fine flour and does not contain ω-5 gliadin [6,8,13]. ω-5 gliadin-deficient wheat is a whole meal flour and was developed by Kohno et al [7].

Acrylamide, bis-acrylamide, urea, acetic acid (17.4 mol L⁻^1^), ammonium persulfate, 2-amino-2-hydroxymethyl-1,3-propanediol (Tris), glycine, sodium dodecyl sulfate (SDS), N,N,N′,N′-tetramethylethylenediamine (TEMED), and 1-propanol were purchased from FUJIFILM Wako Pure Chemical Corporation (Osaka, Japan). Methyl green zinc chloride salt (M8884) was purchased from Sigma-Aldrich (St. Louis, MO, USA). For Coomassie Brilliant Blue (CBB) staining, EzStain AQua (ATTO CORPORATION, Tokyo, Japan) was used.

A 1-mm dual mini gel cast set (AE-6401; 12 lanes, 90 mm (W) × 80 mm (H) × 1 mm (T)), a mini-slab electrophoresis apparatus (AE-6500), and an electrophoresis power supply (AE-8400) were purchased from ATTO CORPORATION. For the electroelution of proteins from the gel band, an agarose gel electrophoresis system (FA-8418; ORIENTAL INSTRUMENTS co., ltd., Kanagawa, Japan) was used.

Step 0: Extraction of gliadin protein fractions from wheat flour.

The gliadin protein fractions were separated using 50% 1-propanol [1,7,10,12]. Wheat flour (2 g) was added to 20 mL of 50% 1-propanol, and the mixture was stirred using a magnetic stirrer (Model M-11, Koike Precision Instruments, Hyogo, Japan) for 1 h at 22 °C. After centrifuging the mixture at 1470 × g for 20 min at 22 °C (S300T; KUBOTA Corporation co., Ltd., Tokyo, Japan), we collected the supernatant. Subsequently, the pellet was re-added to 20 mL of 50% 1-propanol, stirred for 1 h at 22 °C, and centrifuged. Next, the two supernatants were combined, evaporated in vacuo in 37 °C water bath until it was removed 1-propanol (EYELA Rotary Vacuum Evaporator N-N series; TOKYO RIKAKIKAI Co., Ltd., Tokyo, Japan) and lyophilized (FDU-1100, TOKYO RIKAKIKAI Co., Ltd.). The dried material was stored at −20 °C until analysis.

***Step 1: Collection of ω-5 gliadin.***

*Step 1.0: Preparation of A-PAGE gel and gel pre-run.*

Acrylamide A-PAGE gel (8%) preparation and the gel pre-run were conducted according to Watry et al [6]. First, 4 mL of 40% (w/v) acrylamide (29:1 acrylamide:bis-acrylamide), 10 mL ultra-pure water, 2 mL of 50% acetic acid, 0.25 mL of 1.0 mol L⁻^1^ Tris–HCl buffer (pH 8.5), and 0.4 mL of 10% ammonium persulfate were mixed. Thereafter, 6 g of urea was completely dissolved in the mixture, after which the solution was degassed, and 0.2 mL TEMED was added and gently mixed. The gel solution was poured into an empty cassette (90 mm (W) × 80 mm (H) × 1 mm (T)) until full, and the comb was inserted by starting at one end at an angle and slowly lowering it in place to avoid air bubble formation. Thereafter, damp paper towels were placed on top of the cassettes to prevent the solution at the top of the cassette from drying, and the cassettes were incubated at 22 °C for 1 to 2 h. This method yielded two A-PAGE gels.

A-PAGE is run under reverse polarity, with the electrodes connected opposite to those in SDS-PAGE. First, we removed the comb and silicon sticker from the A-PAGE gel cassettes. Afterward, the two gel cassettes were placed in a mini-slab electrophoresis apparatus with the shorter “well” side facing inward, secured with the gel-tension wedge, and the inner chamber was filled with 5% acetic acid as the running solution. The outer chamber was also filled with 5% acetic acid, enough to submerge the bottom edge of the A-PAGE gel cassettes. The lanes of the settled A-PAGE gel were cleaned out by gently pipetting running solution (5% acetic acid). After setting up, the A-PAGE gel was pre-electrophoresed at 22 °C for 90 min at 150 V, or until the milliampere (mA) reading stabilized, ensuring that the pre-run was performed under reverse polarity. After completing the pre-run, the sample can be loaded and the gel run initiated.

*Step 1.1: Gel run and excised ω*-*5 gliadin gel band.*

The gliadin fractions (Step 0) extracted from ‘Harukirari’ and ω-5 gliadin-deficient wheat were dissolved in 4.5 mol L⁻^1^ urea in 5% acetic acid containing methyl green. The crude fraction concentration for ω-5 gliadin collection should be approximately 20 to 50 mg mL⁻^1^ (50 mg mL⁻^1^ was used in this report).

Before loading the samples, the lanes of the set A-PAGE gel were cleaned out by gently pipetting running solution (5% acetic acid). Next, 15 μL of the crude fraction from ω-5 gliadin-deficient wheat was applied to the leftmost lane (Lane 1), and 15 μL of the ‘Harukirari’ crude fraction was applied to the remaining lanes (Lanes 2 to 12). After sample loading, the A-PAGE gel was electrophoresed into ice bath for 3 h at 250 V, ensuring that the gel-run was performed under reverse polarity.

**Fig. 1:**
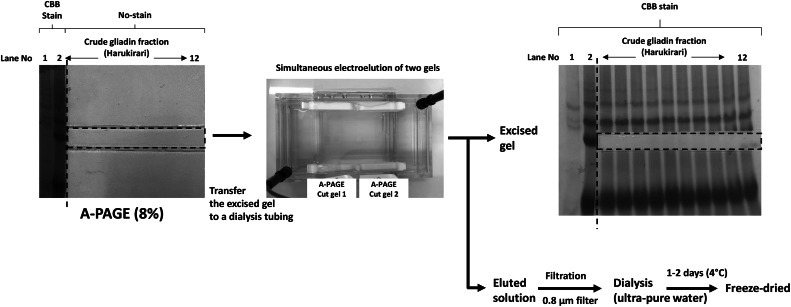
Protein collected from excised gel band using electroelution. In 8% acid-polyacrylamide gel electrophoresis (A-PAGE), the crude fraction from ω-5 gliadin–deficient wheat was applied in the leftmost lane (Lane 1). To the remaining lanes (lanes 2–12), the crude fraction from ‘Harukirari’ was applied.

After the 3-h gel run, the A-PAGE was removed from its cassette, placed on a glass plate, and cut between the second and third lanes from the left (Fig. 1). Thereafter, the shorter gel (lanes 1–2) was subjected to CBB staining for 1 h, whereas the remaining gel (lanes 3–12) was not stained (Fig. 1). Using the shorter stained gel (lanes 1–2) as a reference, the ω-5 gliadin band was excised (approximately 10 mm wide) from the remaining gel (lanes 3–12) without CBB staining (Fig. 1). After excision, the remaining upper and lower gels were subjected to CBB staining (Fig. 1).

*Step 1.2: Protein collection from excised gel band using electroelution.*

The excised ω-5 gliadin gel band was transferred into dialysis tubing (Φ15.9 mm × 25 mm, pore diameter 1.25 nm; molecular weight cutoff: ≈3500 Dalton (Da); AS ONE CORPORATION, Osaka, Japan) with the bottom clipped. The tubing was filled with running solution (5% acetic acid), and the top was clipped to seal it (no air is inside). After submerging the dialysis tubing in an agarose gel electrophoresis system filled with 5% acetic acid, ω-5 gliadin was electroeluted from the excised gel band overnight at 22 °C at 50 V (Fig. 1).

By the next day, the ω-5 gliadin was eluted from the gel band. The eluted solution was transferred from the dialysis tubing and filtered through a 0.8-μm filter (DISMIC-25CS; Toyo Roshi Kaisha, ltd., Tokyo, Japan) to remove gel fragments. Thereafter, the filtered solution was dialyzed against ultra-pure water for 1–2 days at 4 °C and then lyophilized. The gel band was subjected to CBB staining post-electroelution to determine the amount of residual ω-5 gliadin. The freeze-dried powder was dissolved in 150 μL ultra-pure water and stored in a low DNA/protein binding polypropylene tube (BM4015; BM Equipment Co., Ltd., Tokyo, Japan) at −20 °C until analysis.

***Step 2: Recovery rate of ω-5 gliadin, SDS-PAGE, and protein identification.***

*Step 2.0: Preparation of A-PAGE gel and gel pre-run.*

Acrylamide A-PAGE gel (10%) preparation and gel pre-run were performed according to Watry et al [6] (See Step 1.0).

**Fig. 2:**
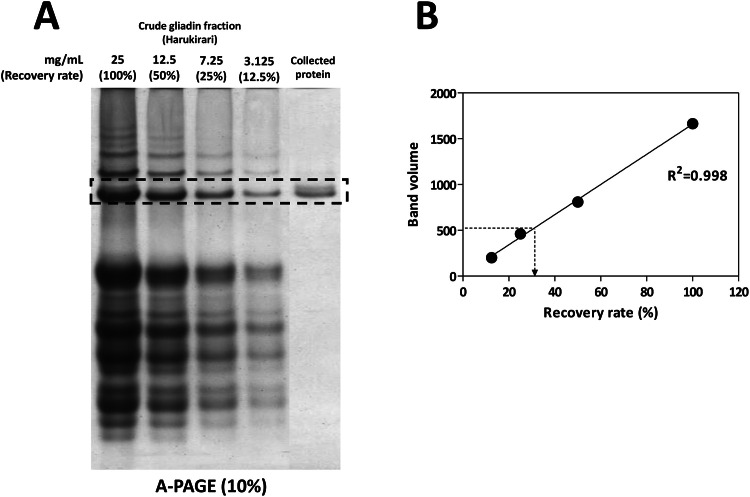
Calculation of recovery rate, A) A-PAGE, B) calibration curve. Crude fractions from ‘Harukirari’ (25, 12.5, 6.25, 3.125 mg mL⁻^1^) and the 2-fold diluted collected ω-5 gliadin solution were subjected to 10% acid-polyacrylamide gel electrophoresis (A-PAGE).

*Step 2.1: Determination of ω*-*5 gliadin recovery rate (*Fig. 2*).*

To prepare a calibration curve for calculating the recovery rate, solutions of the ‘Harukirari’ crude fraction at 25 (corresponding to a recovery rate of 100%), 12.5 (50%), 6.25 (25%), and 3.125 (12.5%) mg mL⁻^1^ were prepared in 4.5 mol L⁻^1^ urea in 5% acetic acid containing methyl green. The collected ω-5 gliadin solution was diluted 2-fold with 4.5 mol L⁻^1^ urea in 5% acetic acid containing methyl green.

Before sample loading, the lanes of the set A-PAGE gel were cleaned out by gently pipetting running solution (5% acetic acid). Thereafter, 15 μL of the ‘Harukirari’ crude fraction and the collected ω-5 gliadin solution were applied, and the A-PAGE gel was electrophoresed into ice bath for 3.5 h at 250 V, ensuring that the pre-run was performed under reverse polarity. After a 3-h gel run, the A-PAGE gel was removed from its cassette and then subjected to CBB staining for 1 h. After 1 h, the stained gel was de-stained in ultra-pure water, repeating the process until the desired level of protein staining was achieved.

The de-stained A-PAGE gel was wrapped with film (Pitatt Clear; ATTO CORPORATION) and scanned using a flatbed scanner (GT-F520; Canon Inc, Tokyo, Japan). Band volume was analyzed using JustTLC software (Sweday, Södra Sandby, Sweden).

*Step 2.2: SDS-PAGE and protein identification.*

SDS-PAGE was performed with 10% polyacrylamide gel (e-PAGEL E-T10l; ATTO CORPORATION) according to the method of Laemmli [14]. The protein molecular-weight standards were supplied in the LMW Marker Kit (Cytiva, Tokyo, Japan), purified ω-5 gliadin was prepared according to Tanaka [9], and SDS-PAGE sample buffer [Sample Buffer Solution without Reducing Reagent(6x) for SDS-PAGE, pH 6.8] was obtained from Nacalai Tesque (Kyoto, Japan). 2-mercaptoethanol (FUJIFILM Wako Pure Chemical Corporation) was used as the reducing agent, and the reduction treatment was conducted in a 70 °C hot-water bath for 10 min.

First, the comb was removed from the SDS-PAGE gel cassette. The gel cassette was then placed in the mini-slab electrophoresis apparatus, and the inner chamber was completely filled with SDS-PAGE running buffer (3 g Tris, 14.4 g glycine, and 1 g SDS in 1 L of water). The outer chamber was filled with SDS-PAGE running buffer, enough to submerge the bottom edge of the SDS-PAGE gel cassette. Before sample loading, the lanes of the set SDS-PAGE gel were cleaned out by gently pipetting SDS-PAGE running buffer. After the reduction treatment, 20 μL of protein molecular weight marker, 1 mg mL⁻^1^ ω-5 gliadin purified using a previously established method [9], and the ω-5 gliadin obtained from the present experiment were applied, and the SDS-PAGE gel was electrophoresed at 22 °C for 50 min at 30 mA. After a 50-min gel run, the SDS-PAGE gel was removed from its cassette and subjected to CBB staining for 1 h. After 1 h, the stained gel was de-stained in ultra-pure water, repeating this process until the desired level of protein staining was achieved. Subsequently, the de-stained SDS-PAGE gel was wrapped in film and scanned using a flatbed scanner. The ω-5 gliadin band obtained using our method was confirmed using nano-liquid chromatography mass/mass spectrometry (nano-LC-MS/MS) analysis.

The nano-LC-MS/MS analysis was modified from previous studies [15,16] and was performed by Japan Proteomics Co. Ltd. (Miyagi, Japan). The ω-5 gliadin band gel was stained by CBB. Gel slippage was reduced by 100 mmol L⁻^1^ of dithiothreitol and alkylated by 100 mmol L⁻^1^ iodoacetamide. After washing, the gels were incubated with trypsin overnight at 30 °C. Recovered peptides were desalted by Ziptip C18 (Merck Millipore, Burlington, MA, USA). Samples were analyzed by nano-LC-MS/MS systems (DiNa HPLC system KYA TECH Corporation/QSTAR XL Applied Biosystems). Mass data acquisitions were piloted by Mascot software (Matrix Science, London, UK).

## Method validation

Wheat gliadins are classified into ω-, γ-, and α/β-gliadins [[1], [2], [3], [4], [5], [6]], of which ω-5 gliadin is a major causative allergen for various types of wheat allergy, including WDEIA [3,[7], [8], [9], [10]]. Conventional methods for purifying ω-5 gliadin are limited by with cost (expensive), laboratory equipment (unpopular) and complexity. Thus, we attempted to develop a cheap and simple method for purifying ω-5 gliadin.

Gliadin classification was based on their mobilities of A-PAGE [[1], [2], [3], [4], [5], [6]] and ω-gliadins are further divided into ω-1,2 gliadin and ω-5 gliadin [[2], [3], [4], [5]]. A-PAGE separates proteins based on their charge and structure; therefore, proteins cannot be estimated from their molecular weight as they are in SDS-PAGE. Thus, distinguishing ω-5 gliadin from the electrophoretic profiles of gliadin fractions on A-PAGE without purified ω-5 gliadin is difficult. To address this, we focused on ω-5 gliadin-deficient wheat flour [2,7,10]. Moreover, some wheat varieties, such as ‘Norin 61,’ lack ω-5 gliadin [6,8,13]. Therefore, we considered that by comparing the A-PAGE profiles of crude gliadin fractions from wheat flour containing ω-5 gliadin with those from wheat flour lacking ω-5 gliadin, identifying the ω-5 gliadin band without using purified protein would be possible.

**Fig. 3:**
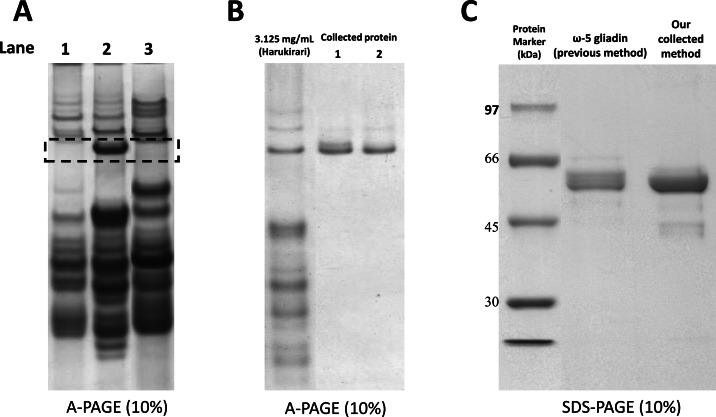
Selection (A) and confirmation (B) of target protein band, and molecular weight analysis of collected proteins (C). In the selection of the target protein band (A), the crude fractions from ω-5 gliadin-deficient wheat [Lane 1 (left)], ‘Harukirari’ [Lane 2 (center)], and ‘Norin 61’ [Lane 3 (right)] were subjected to 10% acid-polyacrylamide gel electrophoresis (A-PAGE). In 10% sodium dodecyl sulfate-polyacrylamide gel electrophoresis (SDS-PAGE) (C), the protein molecular weight marker (left lane), 20 μg lane⁻^1^ ω-5 gliadin purified using a previously established method [7] (center lane), and the ω-5 gliadin collected in the present experiment (right lane) were subjected.

As a result, among the ω-gliadins with low mobility on A-PAGE, we observed a band that was absent in ω-5 gliadin-deficient flour [5] (Fig. 3A). Furthermore, ‘Norin 61,’ a wheat variety that does not contain ω-5 gliadin [6,8,14], did not have a band at the same position (Fig. 3A). Thus, it was suggested that the ω-5 gliadin band was only a present band in the crude gliadin fraction of ‘Harukirari.’ As a preliminary experiment, we attempted to recover proteins by homogenizing the excised gel in 5% acetic acid including 0.1% n-octyl β-d-glucoside and then performing sonication. However, the recovery was very low (data not shown), and we challenged the electroelution. The sample obtained by electroelution was analyzed using A-PAGE, and successful target protein recovery was confirmed (Fig. 3B). The recovery rate (mean ± standard deviation) was 24.5 ± 4.3% (*n* = 4, Fig. 2). SDS-PAGE showed that the ≈55-kDa band in the sample recovered using our method was consistent with that of ω-5 gliadin recovered using the previous method [9] (Fig. 3C). The molecular weight of ω-5 gliadin is reportedly ≈55 kDa [3,8,9]. We also analyzed the most intense SDS-PAGE gel band from the collected sample using nano-LC-MS/MS, and the target band was identified as ω-5 gliadin (Supplementary Table 1).

Finally, we estimated the amount of ω-5 gliadin recovered from ‘Harukirari.’ The crude gliadin fraction obtained from 2.001 g of ‘Harukirari’ was 0.132 g (66.0 mg/g of flour). Moreover, the percentage of ω-5 gliadin in the crude ‘Harukirari’ gliadin fraction was estimated to 9.56% (95.6 mg / g of crude gliadin) by A-PAGE analysis as a reference using previous ω-5 gliadin. Wieser et al. reported that α/β and γ-gliadin were the major components of gliadin, whereas the ω-gliadins occurred in much lower proportions (Min‒Max: ω-5; 4.6‒9.7%, ω-1,2; 5.3‒10.4%, 13 wheat cultivars) of wheat varieties [17]. Based on our results, the recovered amounts of ω-5 gliadin per once experiment and ‘Harukirari’ flour were calculated to 0.176 ± 0.031 mg and 1.55 ± 0.27 mg/g of flour, respectively (*n* = 4).

These results suggest that ω-5 gliadin can be easily purified by combining A-PAGE with electroelution without the need for specialized equipment.

## Limitations

This study had some limitations. The first limitation is that one may not have access to reference flour without ω-5 gliadin. If a reference flour without ω-5 gliadin is unavailable, it may be possible to estimate whether the protein recovered from A-page is ω-5 gliadin by performing SDS-PAGE analysis and confirming that its molecular weight is around 55 kDa.

Second, the recovered sample contained proteins other than ω-5 gliadin. Analysis of the 45-kDa band in SDS-PAGE using nano-LC-MS/MS revealed a low-molecular-weight glutenin subunit (Supplementary Table 2). By utilizing reverse staining (negative staining), one may reduce contamination from proteins other than the target protein. If a more highly purified ω-5 gliadin is required, this method should be combined with gel chromatography or RP-HPLC.

Third, the mini-slab and agarose gel electrophoresis apparatuses are sold by various manufacturers; therefore, procedures such as electroelution must be optimized in each laboratory. Furthermore, we considered that applying this method could enable the recovery of gliadins other than ω-5 gliadin. In this case, the acrylamide concentration in A-PAGE will also need to be examined.

Fourth, the recovered ω-5 gliadin was denatured by urea and therefore not in a fully native state. Watry et al [6] performed A-PAGE without urea, using 5% glycerol as the solvent for the crude gliadin fraction. Consequently, the protein bands appeared more distinct in A-PAGE with urea than without urea [6]. Therefore, whether urea should be used depends on the purpose of the research.

## CRediT authorship contribution statement

**Akihiro Maeta:** Conceptualization, Data curation, Formal analysis, Funding acquisition, Investigation, Methodology, Project administration, Resources, Software, Validation, Visualization, Writing – original draft. **Mamoru Tanaka:** Conceptualization, Methodology, Resources, Validation, Writing – review & editing. **Kyoko Takahashi:** Conceptualization, Funding acquisition, Supervision, Writing – review & editing.

## Declaration of interests

The authors declare that they have no known competing financial interests or personal relationships that could have appeared to influence the work reported in this paper.

## Data Availability

No data was used for the research described in the article.

## Appendix Supplementary materials

Image, application 1
