# Supplementary material for: Development of an inexpensive, simple purification method for ω-5 gliadin from wheat flour
Source: MethodsX. 2026 Jun 1;16:103979. doi: 10.1016/j.mex.2026.103979 (PMC13266216; doi:10.1016/j.mex.2026.103979)
Supplement: Supplementary file 1 [file mmc1.pdf]

Supplementary Table 1. Matching score between the 55-kDa band in SDS-PAGE and ω-5 gliadin [Triticum aestivum] (BAE20328.1) in nano-LC-MS/MS analysis.

Query	Mw_{obs}	Mw_{exp}	Mw_{calc}	Mw_{dif}	Miss	Score [a]	Expect	Rank	Sequences
323	641.9675	1922.8807	1922.9486	-0.0679	0	22	99	1	K.LPQQEFPQQQISQQPQ.Q
332	657.9700	1970.8883	1970.9599	-0.0716	0	36	3.8	1	H.QSPQQQFPQQQFPQQK.L
361	882.7680	2645.2822	2645.3198	-0.0376	0	28	22	1	K.LPQQEFPQQQISQQPQQLPQQQ.Q
391	924.4182	3693.6435	3693.7713	-0.1278	0	38	1.3	1	K.ELHTPQEQFPQQQQFPQPQQFPQQQIPQQH.Q
				Individual ions scores [b]		124			

Mw_{obs}: Observed Molecular weight, Mw_{exp}: Experimental Molecular weight, Mw_{calc}: Calculated Molecular weight, Mw_{dif}: Difference between Mw_{calc} and Mw_{exp}.

[a] Score: Ions score is $-10\times Log_{10}(P)$, where P is the probability that the observed match is a random event.

[b] Individual ions scores > 55 indicate identity or extensive homology ($p<0.05$).

Supplementary Table 2. Matching score between the 45-kDa band in SDS-PAGE and low molecular weight glutenin subunit [Triticum aestivum] (BAD12055) in nano-LC-MS/MS analysis.

Query	Mw_{obs}	Mw_{exp}	Mw_{calc}	Mw_{dif}	Miss	Score [a]	Expect	Rank	Sequences
224	555.2273	1108.4400	1108.5554	-0.1153	0	32	0.79	1	R.VPFGVGTGVGGY.-
390	834.3967	1666.7788	1666.8171	-0.0383	0	38	0.15	1	R.TLPTMCNVNVSLYR.T + Carbamidomethyl (C)
446	692.6703	2074.9892	2075.0292	-0.0401	0	42	0.051	1	K.VFLQQQCSPVAMPQSLAR.S + Carbamidomethyl (C); Oxidation (M)
478	865.6286	3458.4852	3458.5370	-0.0519	0	20	5.3	1	R.SQMLQQSSCHVMQQQCCQQLPQIPQQSR.Y + 3 Carbamidomethyl (C); Oxidation (M)
				Individual ions scores [b]		132			

Mw_{obs}: Observed Molecular weight, Mw_{exp}: Experimental Molecular weight, Mw_{calc}: Calculated Molecular weight, Mw_{dif}: Difference between Mw_{calc} and Mw_{exp}.

[a] Score: Ions score is $-10\times Log_{10}(P)$, where P is the probability that the observed match is a random event.

[b] Individual ions scores > 43 indicate identity or extensive homology ($p<0.05$).
